# Assessment and comparison of phytochemical constituents and biological activities of bitter bean (*Parkia speciosa* Hassk.) collected from different locations in Malaysia

**DOI:** 10.1186/s13065-018-0377-6

**Published:** 2018-02-07

**Authors:** Ali Ghasemzadeh, Hawa Z. E. Jaafar, Mohamad Fhaizal Mohamad Bukhori, Mohd Hafizad Rahmat, Asmah Rahmat

**Affiliations:** 1 Department of Crop Science, Faculty of Agriculture, Universiti Putra Malaysia, 43400 Serdang, Selangor Malaysia; 2 Department of Biology, Universiti Malaysia Sarawak, 94300 Samarahan, Sarawak Malaysia; 3 Department of Nutrition & Dietetics, Faculty of Medicine & Health Sciences, Universiti Putra Malaysia, 43400 Serdang, Selangor Malaysia

**Keywords:** *Parkia speciosa* Hassk, Phytochemicals, DPPH assay, FRAP assay, Antibacterial activity

## Abstract

**Background:**

*Parkia speciosa* seeds are a common ingredient in Malay cuisine with traditional interest because of its medicinal importance and content of health-promoting phytochemicals. This study evaluated the phytochemical constituents and biological activities (antioxidant and antibacterial activities) of *Parkia speciosa* Hassk seeds collected from three different regions of Malaysia (Perak, Negeri Sembilan and Johor). Phytochemical constituents (total flavonoid and total phenolic) were measured using the spectrophotometric method, and individual flavonoids and phenolic acids were identified using ultra-high-performance liquid chromatography. Ferric reducing antioxidant potential (FRAP) assay and 2,2-diphenyl-*1*-picrylhydrazyl (DPPH) assay we used in order to evaluation of antioxidant activities. Disc diffusion method was employed for the evaluation of antibacterial activity of extracts against Gram-positive and Gram-negative bacterial strains.

**Results:**

The primary screening of phytochemicals showed that *P. speciosa* seeds contain alkaloids, terpenoids, flavonoids, and phenolics. Samples collected from Perak contained the highest levels of the phytochemical constituents, with highest DPPH and FRAP activity followed by Negeri sembilan and Johor. From the identified compounds, quercetin and gallic acid were identified as the most abundant compounds. Seeds collected from the Perak location exhibited potent antibacterial activity, against both Gram-positive and Gram-negative bacteria strains. *Staphylococcus aureus* and *Bacillus subtilis* were recorded as the bacterial strains most sensitive to *P. speciosa* seed extracts. Correlation analysis showed that flavonoid compounds are responsible for the antioxidant activities of the *P. speciosa* seeds studied, while antibacterial activity showed a high correlation with the levels of gallic acid.

**Conclusions:**

*Parkia speciosa* seed grown in Perak exhibit the highest concentrations of phytochemicals, as well as the highest biological activity. It may also be recommended for the food industry to use seeds from this area for their products, which are going to compete in the expanding functional food markets.

## Background

Plants present a virtually endless supply of potential cures for humanity. Historically, they have formed the oldest basis for developing medicines used to relieve human suffering and treat many debilitating diseases [1]. A plant can be compared to a chemical factory where a wide range of organic substances is manufactured. Novel bioactive phytochemicals are important feedstock for potential development of new pharmaceuticals and the rich biodiversity of the tropical forest holds great promise for the discovery of such compounds [2]. A major objective of natural product research is the preclinical development of bioactive natural products and their analogues [3]. The production of phytochemicals varies not only between varieties or species but also depends on external variables such as environmental conditions, agricultural practices, and post-harvest handling. Therefore, the phytochemical composition of a given variety/species of plant can vary according to geographic region and this difference can be attributed to geographic differences in type of soil, levels of precipitation, light intensity, humidity, etc. [4, 5].

*Parkia speciosa* Hassk, from the Fabaceae family is a southeast Asian legume. It is locally known in Malaysia as “Patai, Petai” and is generally called “Bitter bean” in English [6]. This plant grows naturally in low land tropical forests and is cultivated in Malaysian villages. The tree grows to a height of 15–40 m, bearing flat, edible bean pods with bright green, plump, almond-shaped seeds [7]. The seeds are flattened and elliptical in shape with a nutty and firm texture. *P. speciosa* seeds are a common ingredient in Malay cuisine and are frequently served beside sambal, dried shrimp, and chili pepper as a popular local delicacy. Several phytochemicals such as flavonoids, phenolics, terpenoids, and fatty acids have been reported in seed extracts of *P. speciosa* [8–11]. In traditional medicine, the seeds of *P. speciosa* are pounded and boiled to be used for alleviating stomach pain and have been considered beneficial in treating liver disease, diabetes, and worm infestations. Besides the culinary uses of *P. speciosa* seed, evidence of anticancer activity [12], antioxidant activity [13], antibacterial activity [14] as well as antiangiogenic activity [13] has been reported by previous studies. Phytochemicals in plants are responsible for their biological activities [4]. Typically, such compounds are produced and accumulate at various levels in plant tissues. Their production strongly correlates to the growing climate, agricultural practices, specific vegetative stages, and other environmental variables [15–17]. Results of previous studies have shown that the production of phytochemicals and the biological activity of the same variety/species of plant can be different when sampling was done from different areas [17]. Therefore, to produce plants with higher phytochemical quality and biological activity it is necessary to optimize the plantation or sampling process. The identification of suitable plantation sites can thus be very important. In Malaysia, it is reported that phytochemical constituents and biological activities of some herbs like as *Murraya koenigii* and *Pandanus amaryllifolius* when sampling was done from different areas [16, 18]. The primary objective of this study was the evaluation and comparison of phytochemical constituents (flavonoids and phenolic acids) and antioxidant and antimicrobial activities of *P. speciosa* extracts from seeds collected in three different plantation sites in the northern, central and southern regions of Malaysia. The correlation between the identified compounds and the biological activity of *P. speciosa* seed extract was also examined.

## Methods

Pod of *P. speciosa* was harvested (at the same time of year in all three regions) from three different locations of Malaysia: Perak in northern Malaysia, Negeri Sembilan in central Malaysia and Johor in southern Malaysia. After cleaning and washing with tap water, the seeds were removed from the pods. Seeds were dried in an oven at temperature of 45 °C for 120 h (5 days). Dried seeds were ground with miller (mesh size 80). Seed powders were kept refrigerated at the temperature of 4–5 °C for future analysis. Samples were submitted to Institute of Bio-science (IBS), Universiti Putra Malaysia and identified as *P. speciosa* Hassk and voucher specimens were deposited at herbarium of IBS.

### Extraction

Five gram of dried seed powder from each sample was transferred to a round-bottom flask. Absolute ethanol (25 mL) was added and the mixture was shaken gently with a shaker at 80 rpm for 10 min. The mixture was then refluxed for 1 h, cooled at room temperature, and filtered using Whatman filter paper No. 1. The solvent was evaporated using a rotary evaporator, and the residue was kept at – 20 °C for future analysis.

### Preliminary screening for phytochemicals

Extracts of *P. speciosa* seeds were subjected to a number of preliminary phytochemical screening tests, as described below. To establish the presence of hydrolyzable tannins, ethanol extracts were treated with a 15% ferric chloride test solution and the resultant color was noted. Blue colour indicated the presence of hydrolyzable tannins. For alkaloid screening, 2 g of each extract were dissolved in 4 mL of ethanol containing 3% tartaric acid. Each test sample was then divided into three test tubes, and tested using Hager’s reagent, Mayer’s reagent, and Marquis reagent. Precipitation in any of the three test tubes indicated the presence of alkaloids. For flavonoid screening, 5 mL of NaOH (20%) were added to each sample of ethanol extract; yellow colour indicated the presence of flavonoids. For phenolic screening, 4 mL of each extract was mixed with water and transferred to a water bath at the temperature of 45 °C. Then, 4 mL of FeCl_3_ (3%) was added. Green or blue colour indicated the presence of phenolic compounds. For saponin screening, 2.5 g of seed powder was extracted with hot water. Then it was cooled to room temperature, shaken vigorously and allowed to stand for 20 min. Froth thickness of more than 1.2 cm indicated the presence of saponins. For terpenoid screening, 1 g of extract was dissolved in 4 mL of chloroform, after which 3 mL H_2_SO_4_ was added. Reddish-brown indicated the presence of terpenoids [19–21].

### Total flavonoid content (TFC)

Crude extracts (5.0 mg) of seeds collected from each of the three locations were dissolved in absolute ethanol (10 mL). For each sample, 5 mL of the resulting solution was mixed with 5 mL of aluminum trichloride solution (2%). Solution was incubated for 10 min in darkness. The absorbance of the solutions was read at 415 nm using a spectrophotometer. For the calibration curve (R^2^ = 995), the absorbance of different concentrations of quercetin (CAS Number 6151-25-3, Sigma-Aldrich, Shah Alam, Malaysia) was read and the final TFC was expressed in milligram quercetin equivalent (QE) per gram dry material (DM) [22, 23].

### Total phenolic content (TPC)

Crude extracts (5.0 mg) of seeds collected from each the three sites were dissolved in ethanol (20 mL each). Afterward, 400 µL of this solution was diluted with 40 mL of distilled water followed by adding 2 mL of Folin–Ciocalteu reagent (tenfold dilution). The mixture was then shaken well and incubated for 10 min in the dark. After incubation, 2 mL of sodium carbonate (7.5%) were added to each sample and the samples were incubated again for 30 min. The absorbance of the samples was read at 765 nm using a spectrophotometer. For the calibration curve (R^2^ = 991), the absorbance of different concentrations of gallic acid (CAS Number 5995-86-8, Sigma-Aldrich, Malaysia)was read and the final TPC was expressed in milligram gallic acid equivalent (GAE) per gram DM [23, 24].

### Identification of individual flavonoids and phenolic acids using UHPLC

Individual flavornoids and phenolic acids were identified using ultra-high-pressure liquid chromatography (UHPLC) with the following specifications: mobile phases were (A) *ortho*-phosphoric acid 0.03 M, (B) Methanol HPLC grade; Column: C18 (5 µm, 4.6 × 250 mm; ZORBAX Eclipse Plus C18), injection volume: 10 µL, flow rate: 1 mL min^−1^, column temperature 35 °C with detector wavelength of 280, 320, and 360 nm. The gradient mode was used as follows: 0 min 4.0%B, 10 min 100%B, 15 min 100%B, and 2.0 min 4.0%B. The injection of each sample and the standards was done in triplicate. The identification of each compound was done by comparing the retention times with standards, UV spectra and UV absorbance ratios after co-injection of samples and standards. All standards were purchase from Sigma-Aldrich (Malaysia).

### Antioxidant analysis

#### 2,2-Diphenyl-*1*-picrylhydrazyl (DPPH) assay

About 6 mL of each seed extract was dissolved in 6 mL of methanolic solution of DPPH (100 µM). The mixture was incubated at 37 °C for 20 min in the dark. The absorbance of the resulting solutions was read at 5.17 nm using a spectrophotometer [22]. α-Tocopherol and butylated hydroxytoluene (BHT) were used as positive controls. The percentage of DPPH activity was calculated as follows:

$$\begin{aligned} {\text{\% inhibition}} & = \\ & \left[ {\left( {{\text{absorbance of control}} - {\text{absorbance of sample}}} \right)/{\text{absorbance of control}}} \right] \times {\text{1}}00 .\\ \end{aligned}$$

#### Ferric reducing antioxidant potential (FRAP) assay

FRAP reagent was prepared fresh as follows: FeCl_3_ (5 mL), 2,4,6-tripyridyl-*S*-triazine (5 mL), acetate buffer (50 mL, pH3.6, 0.3 M L^−1^). The mixture was incubated in a water bath (37 °C) for 20 min in the dark. 1 mL of seed extract was dissolved in 10 mL of FRAP reagent and incubated in a water bath at 26 °C for 30 min in the dark. The absorbance of the solutions was read at 5.93 nm using a spectrophotometer. Acetate buffer was used as the blank. For the standard curve preparation, FeSO_4_·7H_2_O with concentrations ranging from 100 mM to 1000 mM was used. The results were expressed in μM of Fe(II) g^−1^ DM [25].

### Antimicrobial assay

Antibacterial activity of *P. speciosa* seed extracts against Gram-positive and Gram-negative bacteria strains was evaluated using the disc diffusion method. For each sample, 100 mg of crude extract were dissolved in 10 mL of dimethyl sulfoxide (DMSO). Mueller–Hinton agar medium was prepared in Petridishes (15 mL) and sterilized by autoclaving at 120 ± 2 °C for 20 min. After inoculation, the Petri dishes were dried for 15 min. Wells of 6 mm diameter were punched off with a sterile Pasteur pipette and filled with seed extracts (80 µL). The plates were incubated at 37 ± 2 °C for 24 h. Gentamicin and ciprofloxacin at the concentration of 5 µg mL^−1^ were used as a positive control and 10% DMSO was used as a negative control. The zone of inhibition that appeared after 24 h was measured (in mm) as a property of the extract antibacterial activity.

### Evaluation of minimum inhibitory concentration (MIC)

The minimum inhibitory concentration (MIC) of seed extracts was measured by micro dilution assay. A series of diluted extracts (ranging from 20 to 100 µg mL^−1^) were prepared in sterile 96-well micro plates using Mueller–Hinton broth. Bacterial suspension (50 µL) was mixed with an equal volume of each dilution. The blank (150 µL broth) and the bacteria (100 µL broth and 50 µL bacteria suspension) were prepared and gentamicin and ciprofloxacin were used as positive controls. The plates were incubated for 24 h at 37 °C. The diameter of the clear area (in mm) was measured directly on the dishes. The MIC was determined by selecting the lowest concentration (highest dilution) of seed extract that showed no growth of the bacteria strains after 24 h. Three replicates were used for each concentration of the extract (Table 1).

**Table 1:** Climatic and geographical information of sampling area

Locations	Lowest temperature (°C)	Highest temperature (°C)	Above sea level (m)	Average humidity (%)	Average light intensity (µmol m^−2^ s^−1^)	Average sunny day (h)	Average rainfall (mm)
Perak	21	36	45	84	1020	140	224
Negeri Sembilan	22	37	34	80	940	181	195
Johor	23	36	32	78	860	166	181

## Results and discussion

### Preliminary phytochemical screening

The results of the primary phytochemical screening of *P. speciosa* seeds collected from different locations in Malaysia are shown in Table 2. Ethanol extracts of *P. speciosa* seeds collected from Perak, Negeri Sembilan and Johor all contained alkaloids, terpenoids, phenolics, and flavonoids. Saponins and tannins were not observed in any of the *P. speciosa* seed extracts. The presence of phytochemicals in herbs and crops is strongly dependent on the extraction method and solvent type used. These results are consistent with previous studies which showed that chloroform extracts of *P. speciosa* seeds contain terpenoids (e.g., β-sitosterol and stigmasterol) and cyclic polysulfides, namely, hexathionine, tetrathiane, trithiolane, pentathiopane, and pentathiocane [26]. Water and ethanol extracts of *P. speciosa* seeds have also been found previously to contain phenolics (gallic acid) and flavonoids [8, 9].

**Table 2:** Primary screening of phytochemicals from ethanol extract of *P. speciosa* seed

Locations	Alkaloids	Saponins	Terpenoids	Phenolics	Flavonoids	Tannins
Perak	+	−	+	+	+	−
Negeri Sembilan	+	−	+	+	+	−
Johor	+	−	+	+	+	−

+ and − represent presence and absence of compound

### Total flavonoid and individual flavonoid content

Total flavonoid and individual flavonoid content of seed extracts of *P. speciosa* was measured. As depicted in Table 3, TFC varied significantly between the sampled locations. Perak represents the highest TFC (12.4 mg QE g^−1^ DM), followed by Negeri Sembilan (9.2 mg QE g^−1^ DM) and Johor (7.4 mg QE g^−1^ DM). Six distinct flavonoid compounds (quercetin, rutin, kaempferol, catechin, luteolin, and myricetin) were identified from *P. speciosa* seed extracts. High concentrations of quercetin, kaempferol, catechin, luteolin, and myricetin were observed in extracts of seeds harvested in the Perak location. The highest content of rutin was registered at the Negeri Sembilan location. Extracts from the Johor location had low concentrations of all flavonoid compounds, and rutin and kaempferol were not detected in the Johor samples. Several factors influence flavonoid synthesis in herbs and crops, such as environmental conditions (light intensity, CO_2_ concentration, temperature) [27–30], and agricultural practices (fertilizer, irrigation, harvesting, post-harvesting) [31–34]. Wang and Zheng [35] showed that content of flavonoids, phenolics and anthocyanin of strawberry decreased significantly with decreasing of day and night temperature. In a study, Gliszcynska–Swiglo et al. [36] reported a positive and significant correlation between flavonoids content of broccoli and total solar radiation during growth period. Location of plantation was highlighted as a major environmental factor for quercetin content of onion [37]. The differences that this study has found between the sampled locations in TFC and individual flavonoid compounds could be related to environmental conditions such as light intensity, precipitation and temperature levels, and geographical differences. Table 4 show linearity and regression equation of the flavonoid and phenolic compounds.

**Table 3:** Total flavonoid content and some separated flavonoid compounds from ethanol extract of *P. speciosa* seed collected from different locations of Malaysia

Locations	Total flavonoids	Quercetin	Rutin	Kaempferol	Catechin	Luteolin	Myricetin
Perak	12.4 ± 3.51^a^	2.71 ± 0.69^a^	1.80 ± 0.29^a^	0.66 ± 0.09^a^	1.48 ± 0.59^a^	1.00 ± 0.19^a^	0.76 ± 0.22^a^
Negeri Sembilan	9.2 ± 1.49^b^	2.15 ± 0.49^a^	1.91 ± 0.38^a^	0.42 ± 0.04^b^	1.15 ± 0.24^a^	0.66 ± 0.05^b^	0.27 ± 0.02^c^
Johor	7.4 ± 1.88^c^	1.47 ± 0.38^b^	ND	ND	0.90 ± 0.33^b^	0.49 ± 0.01^c^	0.42 ± 0.03^b^

Data are means of triplicate measurements ± standard deviation. Means not sharing a common single letter in each column for each measurement were significantly different at P < 0.05. The units of total flavonoids and flavonoid compounds are mg quercetin equivalents per gram DM and mg per gram DM

*ND* not detected

**Table 4:** Linearity and regression equation of the flavonoid and phenolic compounds

Compounds	UV (λ_max_)	R_t_ (min)	Linear regresion	R^2^	LOD (µg mL^−1^)	LOQ (µg mL^−1^)
Quercetin	355	10.2	y = 92.846x + 37.26	0.9991	0.91	3.02
Rutin	260	4.8	y = 86.437x + 22.71	0.9984	1.20	3.98
Kaempferol	275	18.7	y = 146.209x + 30.61	0.9947	0.67	2.24
Catechin	280	3.9	y = 452.017x + 62.19	0.9996	0.16	0.53
Luteolin	275	14.5	y = 265.733x + 46.52	0.9993	0.30	0.99
Myricetin	275	12.8	y = 109.357x + 59.34	0.9957	0.83	2.82
Gallic acid	280	2.6	y = 864.620x-114.17	0.9928	0.05	0.18
Ferulic acid	320	6.4	y = 640.052x + 88.14	0.9991	0.12	0.39
Caffeic acid	280	3.8	y = 261.55x + 56.20	0.9970	0.28	0.93
trans-Cinnamic acid	280	4.7	y = 173.062x + 44.91	0.9994	0.58	1.91
*p*-coumaric acid	320	11.1	y = 243.526x + 84.28	0.9961	0.34	1.13

*R*_*t*_ retention time, *y* peak area, *x* concentration of standard (µg mL^−1^), *R*^*2*^ correlation coefficient for six data point in the calibration carve (n = 3), *LOD* limit of detection, *LOQ* limit of quantification

### Total phenolic and individual phenolic acid content

Total phenolic and individual phenolic acid content from seed extracts of *P. speciosa* was measured. As demonstrated in Table 5, TPC was significantly influenced by sampling location. The highest TPC was recorded at Perak (26.3 mg GAE g^−1^ DM) followed by Negeri Sembilan (20.5 mg GAE g^−1^ DM) and Johor (14.9 mg GAE g^−1^ DM). Five phenolic acids (gallic acid, caffeic acid, ferulic acid, trans-cinnamic acid, and *p*-coumaric acid) were identified. In a result similar to that of the flavonoid assay, Perak had the highest concentration of phenolic acids followed by Negeri Sembilan and Johor. Caffeic acid was not detected in the seed extracts from Negeri Sembilan, and no significant difference was observed between Perak and Johor samples in caffeic acid content. Ferulic acid and *p*-coumaric acid were not detected in the Johor samples either

**Table 5:** Total phenolic content and some separated phenolic compounds from ethanol extract of *P. speciosa* seed collected from different locations of Malaysia

Locations	Total phenolics	Gallic acid	Caffeic acid	Ferulic acid	trans-cinnamic acid	*p*-coumaric acid
Perak	26.3 ± 2.74^a^	6.42 ± 0.67^a^	1.46 ± 0.67^a^	2.71 ± 0.89^a^	1.84 ± 0.45^a^	2.73 ± 0.41^a^
Negeri Sembilan	20.5 ± 2.26^b^	5.11 ± 0.59^b^	ND	2.26 ± 0.83^a^	1.05 ± 0.29^b^	1.89 ± 0.32^b^
Johor	14.9 ± 2.03^c^	3.56 ± 0.28^c^	1.19 ± 0.37^a^	ND	0.64 ± 0.04^c^	ND

Data are means of triplicate measurements ± standard deviation. Means not sharing a common single letter in each column for each measurement were significantly different at P < 0.05. The units of total phenolics and phenolic compounds aremg gallic acid equivalents per gram DM and mg per gram DM

*ND* not detected

### Antioxidant activity

Ethanol extracts of *P. speciosa* seed collected from the three locations were evaluated for antioxidant activity using DPPH and FRAP assays. As shown in Table 6, DPPH free radical scavenging activity of extracts was influenced significantly by the sampling location. The highest activity was observed in the extract from the Perak site (66.29%) followed by Negeri Sembilan (52.47%) and Johor (41.62%). DPPH activity of all extracts was lower than the positive standards (α-tocopherol = 84.19% and BHT = 70.58%). From the sampled sites, Perak exhibited lowest IC_50_ (the half-maximal inhibitory concentration) value (86.7 µg mL^−1^) and Johor exhibited highest IC_50_ content (153.1 µg mL^−1^). Lower IC_50_ values represent stronger free radical inhibition, as strong free-radical inhibitors are active at low concentrations. The ranking order of FRAP activity was as follows: Perak (522.1 μM of Fe(II) g^−1^), followed by Negeri Sembilan (462.5 μM of Fe(II) g^−1^), followed by Johor (407.5 μM of Fe(II) g^−1^). The lowest IC_50_ value was seen in the extracts from the Perak location (91.5 µg mL^−1^), followed by Negeri Sembilan (121.2 µg mL^−1^) and Johor (140.6 µg mL^−1^). α-Tocopherol showed FRAP activity, which was higher than that of the *P. speciosa* seed extracts at all three locations. More interestingly, the FRAP activity of Perak extracts was higher than BHT, but no significant differences were observed between the extracts from the Perak location and BHT. Several studies reported that the antioxidant activity of herbs is significantly associated with their phytochemical content, especially that of flavonoids and phenolic acids [38–40]. In this study, the highest antioxidant activity as well as the highest content of flavonoids and phenolic acids was observed in *P. speciosa* seed extracts from the Perak location. Alternatively, variation in climatic conditions, soil nutrients, water quality (hydrogen potential, electrical conductivity), and agricultural activity could influence the production of phytochemicals, which in turn could affect the antioxidant activities.

**Table 6:** DPPH and FRAP scavenging activities (at concentration of 100 µg mL^−1^) and IC_50_ value of ethanol extract of *P. speciosa* seed collected from different locations of Malaysia

Locations	DPPH free radical scavenging activity (%)	IC_50_ (µg mL^−1^)	Ferric reducing antioxidant potential (μM of Fe(II) g^−1^)	IC_50_ (µg mL^−1^)
Perak	66.29 ± 4.88^b^	86.7 ± 5.80^c^	522.1 ± 18.29^b^	91.5 ± 7.83^c^
Negeri Sembilan	52.47 ± 4.46^c^	109.2 ± 6.12^b^	462.5 ± 14.80^c^	121.2 ± 7.14^b^
Johor	41.62 ± 2.71^d^	153.1 ± 6.32^a^	407.5 ± 11.62^d^	140.6 ± 8.49^a^
Positive controls				
α-tocopherol	84.19 ± 5.20^a^	42.6 ± 3.25^e^	871.2 ± 20.48^a^	44.9 ± 3.91^e^
BHT	70.58 ± 4.35^b^	79.6 ± 4.04^d^	514.5 ± 15.20^b^	93.5 ± 4.37^c^

Data are means of triplicate measurements ± standard deviation. Means not sharing a common single letter in each column for each measurement were significantly different at P < 0.05

*No* represent not observed

### Antibacterial activity

The antibacterial activity of *P. speciosa* seed extracts collected from different locations in Malaysia against both Gram-positive and Gram-negative bacteria is shown in Table 7. The antibacterial activity was significantly influenced by the sampling location. Extracts from the Perak location had a strong inhibitory effect on all Gram-positive and Gram-negative bacterial strains tested, followed by extracts from Negeri Sembilan and Johor. Among the bacterial strains used, *Bacillus subtilis* was the most sensitive to *P. speciosa* seed extracts. Extracts from Negeri Sembilan and Johor did not show antibacterial activity against *Listeria monocytogenes.* The Johor seed extracts also did not show antibacterial activity against *Pseudomonas aeruginosa.* Seed extracts from all three locations had a lower antibacterial effect than gentamicin and ciprofloxacin, which were used as positive controls. Generally, results showed that Gram-positive bacteria are more sensitive to *P. speciosa* extracts than Gram-negative bacteria. A recent study showed that the pod extract of *P. speciosa* also exhibits antibacterial activity against *Bacillus cereus, L. monocytogenes, S. aureus,* and *Escherichia coli*, with inhibition ranging 6.87 and 11.50 mm [41]. Gram-negative bacteria possess an outer membrane surrounding the cell wall, which restricts the diffusion of hydrophobic compounds through its lipopolysaccharide covering. Without an outer membrane, the extract is able to disrupt the cytoplasmic membrane, causing increased cell wall and cell membrane permeability. Moreover, it can disrupt the proton motive force, electron flow, active transport and coagulation of cell contents [42]. Our findings in this study are consistent with Musa et al. who reported that Gram-positive bacteria showed mostly sensitivity to *P. speciosa* extract, while Gram-negative bacteria were resistant to it [43]. The minimal inhibitory concentration (MIC) of seed extracts from the three different locations ranged between 40 and 100 µg mL^−1^ (Table 8). A lower MIC value indicates stronger antibacterial activity, as strong bacterial inhibitors are active at low concentrations. Therefore, *S. aureus* was sensitive to seed extracts from Perak to *Bacillus subtilis* was sensitive to seed extracts from both Perak and Negeri Sembilan, with MIC of 40 µg mL^−1^.

**Table 7:** Antibacterial activity of ethanol extract of *P. speciosa* seed collected from different locations of Malaysia and antibiotics against bacterial strains

Bacterial strains	Inhibition zone (mm)					
	Perak	Negeri Sembilan	Johor	Gentamicin	Ciprofloxacin	DMSO: water (1:9 v/v)
*S. aureus*	7.2 ± 0.346^b^	5.1 ± 0.340^c^	5.0 ± 0.462^c^	8.4 ± 0.401^a^	7.4 ± 0.328^b^	No
*B. subtilis*	8.4 ± 0.320^b^	8.2 ± 0.411^b^	6.2 ± 0.140^c^	9.3 ± 0.355^a^	8.0 ± 0.349^b^	No
*L. monocytogenes*	2.0 ± 0.151^c^	No	No	4.0 ± 0.307^b^	4.5 ± 0.279^a^	No
*E. coli*	1.7 ± 0.130^c^	1.2 ± 0.153^d^	0.5 ± 0.115^e^	4.7 ± 0.227^a^	4.0 ± 0.201^b^	No
*S. typhimurium*	5.6 ± 0.429^c^	4.1 ± 0.208^d^	5.3 ± 0.346^c^	6.8 ± 0.430^a^	6.0 ± 0.490^b^	No
*P. aeruginosa*	4.1 ± 0.283^b^	2.8 ± 0.116^c^	No	5.4 ± 0.461^a^	5.1 ± 0.406^a^	No

All analyses are the mean of triplicate measurements ± standard deviation. Means not sharing a common letter in each row were significantly different at P < 0.05

*No* not observed

**Table 8:** Minimal inhibitory concentration (MIC) of ethanol extract of *P. speciosa* seed collected from different locations against bacterial strains

Bacterial strains	Perak	Negeri Sembilan	Johor
*S. aureus*	40.0	80.0	80.0
*B. subtilis*	40.0	40.0	60.0
*L. monocytogenes*	> 100	No	No
*E. coli*	> 100	> 100	> 100
*S. typhimurium*	80.0	> 100	80.0
*P. aeruginosa*	80.0	> 100	No

All analyses are the mean of triplicate measurements ± standard deviation; unit is µg mL^−1^

*No* not observed

### Correlation analysis

It is important to examine the correlations between the phytochemical content and the biological activity of crops or herbs in order to identify the compounds responsible for the biological activity of each plant. This knowledge could help researchers to establish the most suitable growth conditions and the best harvesting and extraction techniques in order to maximize the production of the compounds of interest. In this study, correlation analysis between identified phytochemicals and biological activities of *P. speciosa* seed was examined (Table 9). The DPPH activity of *P. speciosa* seed extracts was found to be significantly correlated with flavonoid and phenolic acid content, with the exception of caffeic acid (R^2^ = 0.525) and *p*-coumaric acid (R^2^ = 0.619). The highest correlation was seen between DPPH activity and TFC (R^2^ = 0.941). In the FRAP analysis, FRAP activity also correlated significantly with flavonoid and phenolic acid content, with the exceptions of ferulic acid, caffeic acid and *p*-coumaric acid. The highest correlation was seen between FRAP activity and TFC (R^2^ = 0.966). Antibacterial activity also correlated significantly with flavonoids and phenolic acids, except rutin, caffeic acid, and *p*-coumaric acid. The highest correlation was seen between antibacterial activity and TPC (R^2^ = 0.933). Our findings in current study are consistent with those of previous studies, which have shown positive and significant correlations between flavonoid and phenolic acid levels and the biological activity in herbs and crops [39, 40, 44]. The chemical diversity of plants is more complex than any chemical library made by humans, and the plant kingdom therefore represents an enormous reservoir of valuable molecules just waiting to be discovered.

**Table 9:** Correlation analysis between identified phytochemicals and biological activities of *P. speciosa* seed

Phytochemicals	DPPH activity	FRAP activity	Antibacterial activity
TFC	0.941**	0.966**	0.906**
TPC	0.883**	0.860**	0.933**
Quercetin	0.911**	0.894**	0.917**
Rutin	0.728*	0.741*	0.611 ^n.s^
Kaempferol	0.930**	0.862**	0.889**
Catechin	0.886**	0.841**	0.847**
Luteolin	0.886**	0.900**	0.895**
Myricetin	0.820**	0.871**	0.755*
Gallic acid	0.900**	0.844**	0.921**
Ferulic cid	0.749*	0.669 ^n.s^	0.882**
Caffeic acid	0.525^n.s^	0.627^n.s^	0.600 ^n.s^
trans-Cinnamic acid	0.861**	0.794*	0.781*
*p*-coumaric acid	0.619^n.s^	0.406^n.s^	0.473^n.s^

n.s, * and ** represent non-significant, significant at p < 0.05 and p < 0.01, respectively

## Conclusion

The results of this study indicate that the phytochemical composition and the biological activity of *P. speciosa* seeds vary significantly depending on where in Malaysia it is grown. *P. speciosa* grown in the Perak displayed the highest phytochemical content, antioxidant and antibacterial activities. They were followed by the Negeri Sembilan and Johor regions. The extracts contained substantial amounts of quercetin, kaempferol, and gallic acid, all of which potently inhibited the growth of Gram-positive and Gram-negative bacteria. The biological activity of *P. speciosa* seed extracts significantly correlated with their flavonoid content, followed by the phenolic acid content. The results of this study strongly suggest using the Perak location for plantation and sampling of *P. speciosa* and for further investigation.

## Abbreviations

DMSO: dimethyl sulfoxide
DPPH: 2,2-diphenyl-1-picrylhydrazyl
IC_50_: half-maximal inhibitory concentration
MTT: (3-(4,5-dimethylthiazol-2-yl)-2,5-diphenyltetrazolium bromide)
TFC: total flavonoid content
TPC: total phenolic content
UHPLC: ultra-high performance liquid chromatography
